# Lyotropic Aqueous 2-Picolinium Ionic Liquid Crystals and Their Shear-Induced Foams

**DOI:** 10.1021/acs.langmuir.4c02059

**Published:** 2024-08-17

**Authors:** Andreia F. M. Santos, Anton Gradišek, Tomaž Apih, Pedro J. Sebastião, Madalena Dionísio, Luis C. Branco, João L. Figueirinhas, Maria H. Godinho

**Affiliations:** † LAQV-REQUIMTE, Department of Chemistry, NOVA School of Science and Technology, NOVA University of Lisbon, Campus de Caparica, 2829-516 Caparica, Portugal; ‡ Jožef Stefan Institute, Jamova Cesta 39, 1000 Ljubljana, Slovenia; § CeFEMA and Department of Physics, Instituto Superior Técnico, University of Lisbon, Av. Rovisco Pais, 1, 1049-001 Lisbon, Portugal; ∥ i3N/CENIMAT, Department of Materials Science, NOVA School of Science and Technology, NOVA University of Lisbon, Campus de Caparica, 2829-516 Caparica, Portugal

## Abstract

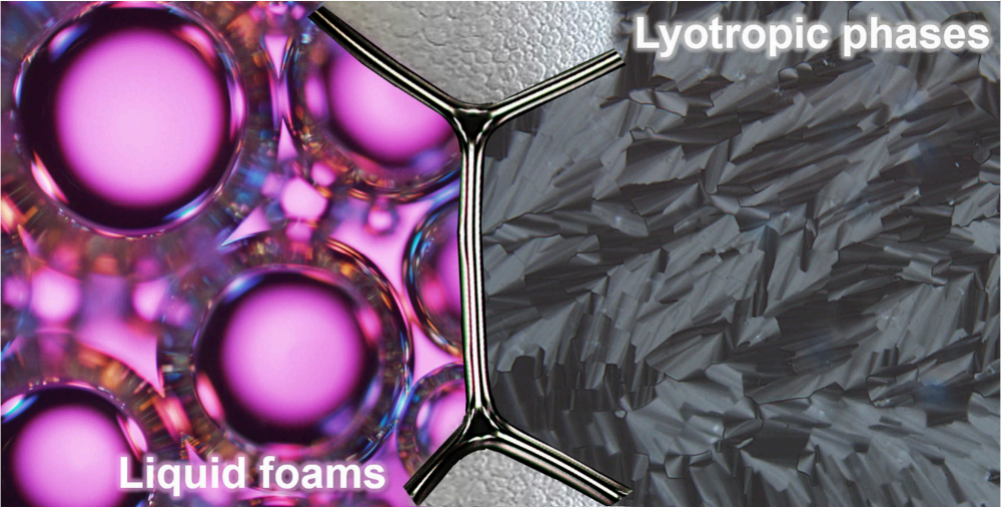

1-Dodecyl-2-methylpyridinium bromide ([C_12_-2-Pic][Br]) and 1-hexadecyl-2-methylpyridinium bromide ([C_16_-2-Pic][Br]) are two ionic liquid crystals presenting thermotropic smectic phases above 80 °C. Aiming to take advantage of the liquid crystalline properties at lower temperatures, lyotropic aqueous systems were prepared from these two organic salts. Both systems were characterized by polarized optical microscopy (POM), X-ray powder diffraction (XRD), and fast field cycling nuclear magnetic resonance (FFC-NMR) relaxometry to assess their texture, phase structure, and molecular dynamics, respectively. The mesomorphic behavior was induced at room temperature. Moreover, the lyotropic [C_12_-2-Pic][Br]_aq_ revealed a smectic phase with higher separation between layers, different from the lamellar phases found in the thermotropic system (S_1_ and S_A_), which is thermally stable up to 50 °C. Furthermore, the surfactant nature of the ionic liquids diluted solutions investigated in this work allowed the formation of foams. It was found that the precursor solutions of the lyotropic dilutions with the longest alkyl chain ([C_16_-2-Pic][Br]_aq_) originated liquid foams with more stable structures than those of [C_12_-2-Pic][Br]_aq_.

## Introduction

Lyotropic systems are ubiquitous in nature, being found mainly in aqueous media. In these liquid crystalline mixtures, the mesophase emerges when an amphiphilic mesogen is dissolved in a suitable solvent and the concentration is enough to promote the formation of molecular aggregates, known as micelles.^1−5^ There are some examples of lyotropic ionic liquid crystals described in the literature.^6^ Regarding their phase structure, a hexagonal phase was reported for the aqueous solutions based on the methylimidazolium surfactants [C_10_MiM][NO_3_],^7^ [C_14_MiM][Br],^8^ and [C_16_MiM][Acr],^9^ in which the latter also displays cubic phases.^9^ In contrast, metal alkanoates dissolved in water exhibit smectic mesophases.^10−12^ Additionally, these phases were also observed for protic pyridiniums^13^ and for anionic surfactant carboxylates^14^ in the presence of various solvents. It is worthwhile mentioning that, for choline laurate [Ch][Lau], in dimethyl sulfoxide (DMSO), a transition from hexagonal to a lamellar phase was detected upon the addition of α-tocopherol.^14^ In another approach, ternary phase diagrams obtained from mixtures of a nonionic surfactant in water and [C_4_MiM][BF_4_] or [C_4_MiM][PF_6_] revealed different mesophases: lamellar, hexagonal, and cubic.^15^ Furthermore, the surfactant nature of the lyotropic constituent molecules can be associated with the presence of foams.^16,17^ There are several parameters involving the bulk solution and interfacial properties that affect the foam dynamics and stabilization: viscosity (bulk and surface), surface tension, and rotation speed, among others.^18^ The presence of these colloidal species influences the production and applicability of lyotropic phases.^16^ Moreover, foams themselves have also a wide range of potential uses, including the production of fibers,^19^ where foam formation is extremely relevant, rechargeable batteries,^20^ and stimuli-responsive materials,^21^ as well as delivery systems for pharmaceutical applications^22^ and porous media for oil recovery.^23^ Notably, their appearance was registered for ionic liquids crystals (ILCs).^24,25^ The resulting morphologies revealed to be reminiscent of two-dimensional liquid foams, in which the material was seen to partition into dark domains (henceforth termed “cells” or “bubbles”) separated by brighter and birefringent walls that are approximately arcs of circles and meet at the vertices (“plateau borders”) with three or more sides.^24,25^

Recently, we reported several ionic liquid crystals based on pyridinium and picolinium cations.^26,27^ These low melting organic salts exhibit thermotropic smectic phases within different temperature ranges. Among these ILCs, 1-dodecyl-2-methylpyridinium bromide ([C_12_-2-Pic][Br]) and 1-hexadecyl-2-methylpyridinium bromide ([C_16_-2-Pic][Br]) are the ones transiting to liquid crystal at higher temperatures. Therefore, aiming to bring the mesophase close to room temperature, potentially lyotropic aqueous systems were developed. In this context, there is a crucial aspect to be considered: the concentration, as overly diluted molecules are not able to aggregate and form liquid crystalline structures. On the contrary, the presence of a small amount of water might not be enough to disturb the positional order addressed to crystalline materials. Thus, it is important to determine the critical concentration, i.e., the concentration above which the mesomorphic behavior arises. Additionally, diluted solutions were prepared to determine the critical micelle concentration (CMC), a parameter directly related to foam formation. The film morphologies associated with the foams of [C_12_-2-Pic][Br]_aq_ and [C_16_-2-Pic][Br]_aq_ bear a very strong resemblance to those of two-dimensional foams already mentioned.^24,25^ To the best of our knowledge, no studies have been published combining lyotropic ionic liquid crystals with the development of foams.

Herein, both picolinium derivatives were investigated in aqueous solutions, showing lyotropic arrangements with no need for an extra LC constituent. These phases were studied by polarized optical microscopy (POM), X-ray powder diffraction (XRD), and fast field cycling nuclear magnetic resonance (FFC-NMR) relaxometry. As smectic liquid crystalline phases have optical birefringence and are characterized by both orientational and position order, POM and XRD allow to evaluate their optical and structural properties. Regarding NMR, it conveys information about the molecular dynamics of the system.^28^ In particular, the spin–lattice relaxation rate, *R*_1_, is an NMR observable property that measures molecular motions at different time and length scales. As some of the motions are intimately related to the structure of the mesophase, *R*_1_ frequency dispersions can provide relevant insights into the characterization of liquid crystalline materials.^28,29^

Furthermore, less concentrated mixtures were also prepared to determine the CMCs through electrical conductivity measurements. Finally, the analysis of the foams associated with each compound was performed by POM as a function of time, evaluating the kinetics addressed to the foam disappearance.

## Experimental Part

### General Remarks

All commercial organic solvents were purchased from different chemical companies, whereas bromoalkanes and 2-methylpyridine were supplied by Sigma-Aldrich (97% purity) and Solchemar (98% purity), respectively.

### Synthesis of Ionic Liquid Crystals

1-Dodecyl-2-methylpyridinium bromide ([C_12_-2-Pic][Br]) and 1-hexadecyl-2-methylpyridinium bromide ([C_16_-2-Pic][Br]) were previously synthesized and chemically characterized.^26,27^

### Preparation of Lyotropic Systems

For both ILCs, several potentially lyotropic formulations, with different concentrations in terms of weight, were developed by dissolving [C_12_-2-Pic][Br] or [C_16_-2-Pic][Br] in distilled water. The aqueous solutions were prepared ensuring their homogeneity. Therefore, the mixtures containing [C_12_-2-Pic][Br] range from 10 to 60 wt %. For the salt with the longer chain, it was only possible to prepare concentrations up to 40 wt %.

### Foam Formation

The foams studied in this work were derived from the isotropic solutions prepared to measure the critical micelle concentration. In this context, each vial was shaken manually, and the resulting foams were transferred to a glass slide and pressed with a coverslip for further observation under POM.

### Characterization of Lyotropic Ionic Liquid Crystals and Their Foams

#### Polarized Optical Microscopy (POM)

POM images were obtained in transmission mode, between crossed polarizers, using an Olympus BX51 microscope coupled with an Olympus DP73 CCD camera and acquired with the Stream Basic v.1.9 Olympus software. A cold illumination source generated by a halogen lamp (Olympus, model KL 2500 LCD) was used. The images were obtained with ×10 or ×20 objectives (Olympus, MPlanFL N) and automatically scaled by the software. For foam studies, some microphotographs were collected with a retardation waveplate (λ = 530 nm) placed at 45°.

#### X-ray Powder Diffraction (XRD)

X-ray scattering profiles were acquired using the powder method and a variable geometry setup equipped with a Max-Flux Optic graded multilayer monochromator (Cu Kα radiation: λ = 1.54056 Å) and an INEL CPS 590 gas curved detector in association with a computer-controlled data acquisition system. The apparatus acquisition system was calibrated by using the scattering peaks of a silver behenate sample. Data were analyzed on Peakoc Application Version 1.0 supplied by INEL.

#### Nuclear Magnetic Resonance (NMR) Relaxometry

NMR relaxation dispersions were measured on a 7 T (300 MHz for ^1^H) Bruker NMR spectrometer using the conventional inversion–recovery radio-frequency pulse sequence and, in the frequency range from 10 kHz to 18 MHz, a Stelar Spinmaster 0.5 T relaxometer through the standard nonpolarized and prepolarized routines fast field cycling (FFC) NMR techniques. Time domain signals were collected above 6 MHz for the first protocol and below this value for the second one. The polarization frequency of the latter was 18 MHz, which corresponds to 0.42 T. Moreover, the acquisition frequency in both routines was 9.25 MHz. It was found that the magnetization decays obtained for both FFC and conventional inversion–recovery experimental techniques were biexponential with two *R*_1_ rates. The component with the smallest value was assigned to bulk water contribution. In this work, the proton spin–lattice relaxation rates of the largest component were quantitatively analyzed by fitting a relaxation model to the data using a nonlinear least-squares minimization procedure. The model comprises the most important relaxation mechanisms often found in mesomorphic systems: (i) translational self-diffusion (SD), modulating intermolecular magnetic dipolar proton interactions, (ii) director fluctuations, specifically layer undulations (LU), which are expected to be effective at low frequencies, and (iii) molecular rotations or reorientations (Rot), reflecting intramolecular proton spin dipolar interactions. Considering that these relaxation mechanisms are statistically independent, the obtained relaxation rates (*R*_1_) can be expressed as a function of the Larmor frequency, υ, by eq 1:^28,30,31^
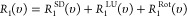
1Rotations and self-diffusion relaxation mechanisms are the main contributors to the *R*_1_ dispersion profile at frequencies above 10^5^ Hz. Layer undulations, on the other hand, can be responsible for the increase of *R*_1_ in the low frequency range dispersion profile, depending on the viscoelastic properties of the materials and the coherence length associated with the lamellar structure addressed to smectic phases. Furthermore, this increase is characterized by the distinct power law log(*R*_1_) ∼ −υ.

#### Critical Micelle Concentration (CMC)

The CMC values of [C_12_-2-Pic][Br] and [C_16_-2-Pic][Br] were extrapolated by the electrical conductivity method. Several aqueous solutions were prepared with different concentrations: from 2 to 20 mM in the case of [C_12_-2-Pic][Br]_aq_ and between 0.1 and 5 mM for [C_16_-2-Pic][Br]_aq_. First, water conductivity was measured as a reference. Then, the experiments were conducted without stirring on a Crison Basic30 EC Meter conductometer coupled to a Crison Pt 1000 electrode. The micellization profiles of both materials were constructed with the average of four independent electrical conductivities acquired for each solution against the concentration. The respective values are in Table S1.

## Results and Discussion

### Mesomorphic Behavior of Lyotropic Systems

**Figure 1:**
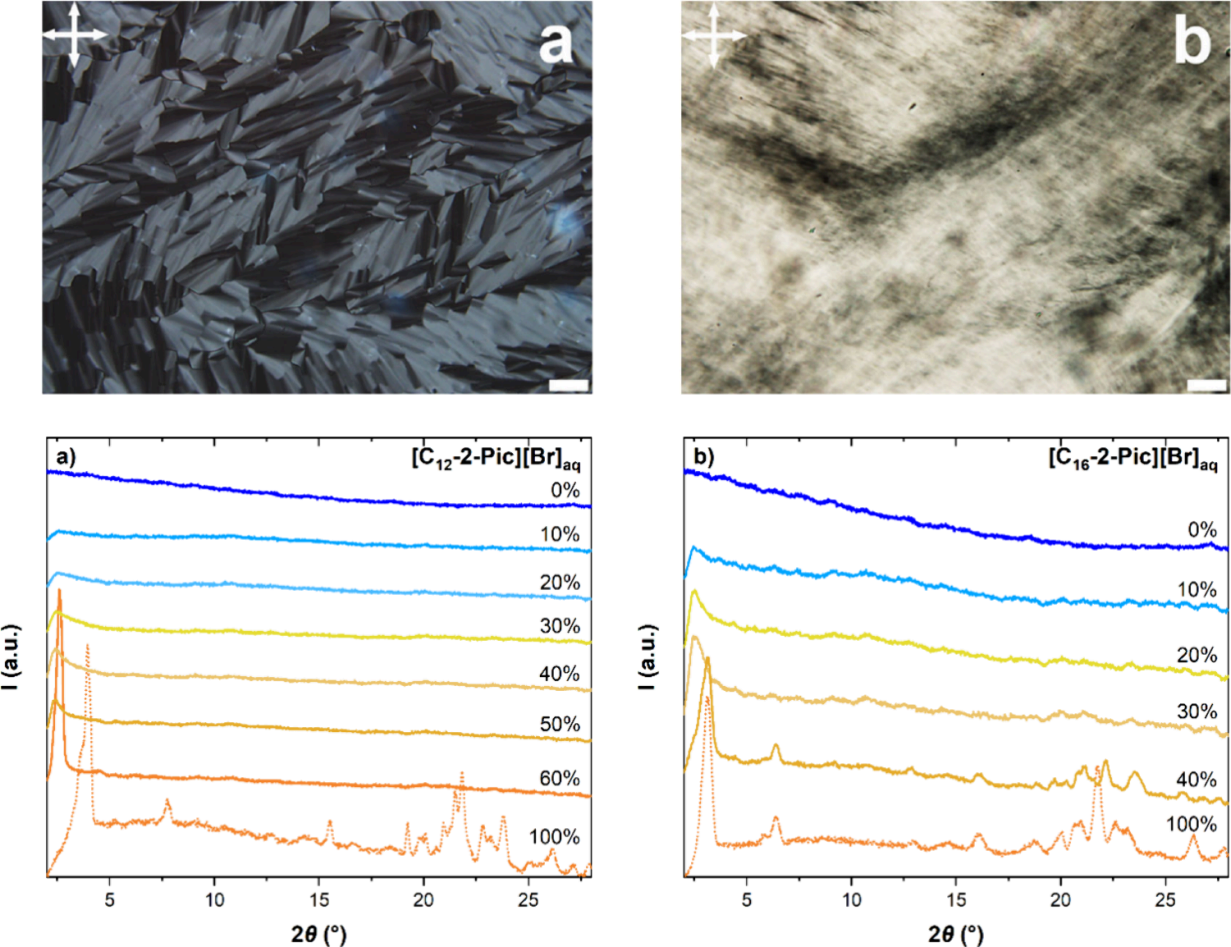
Optical microphotographs (top) and diffractograms (bottom) obtained for (a) [C_12_-2-Pic][Br]_aq_ and (b) [C_16_-2-Pic][Br]_aq_. POM images were acquired between crossed polarizers at the highest concentration prepared: 60% and 40%, respectively. Data were collected at room temperature, with the exception of the [C_16_-2-Pic][Br]_aq_ POM that was taken at 30 °C, where birefringent domains were observed after shearing. The scale bars indicate 50 μm.

**Figure 2:**
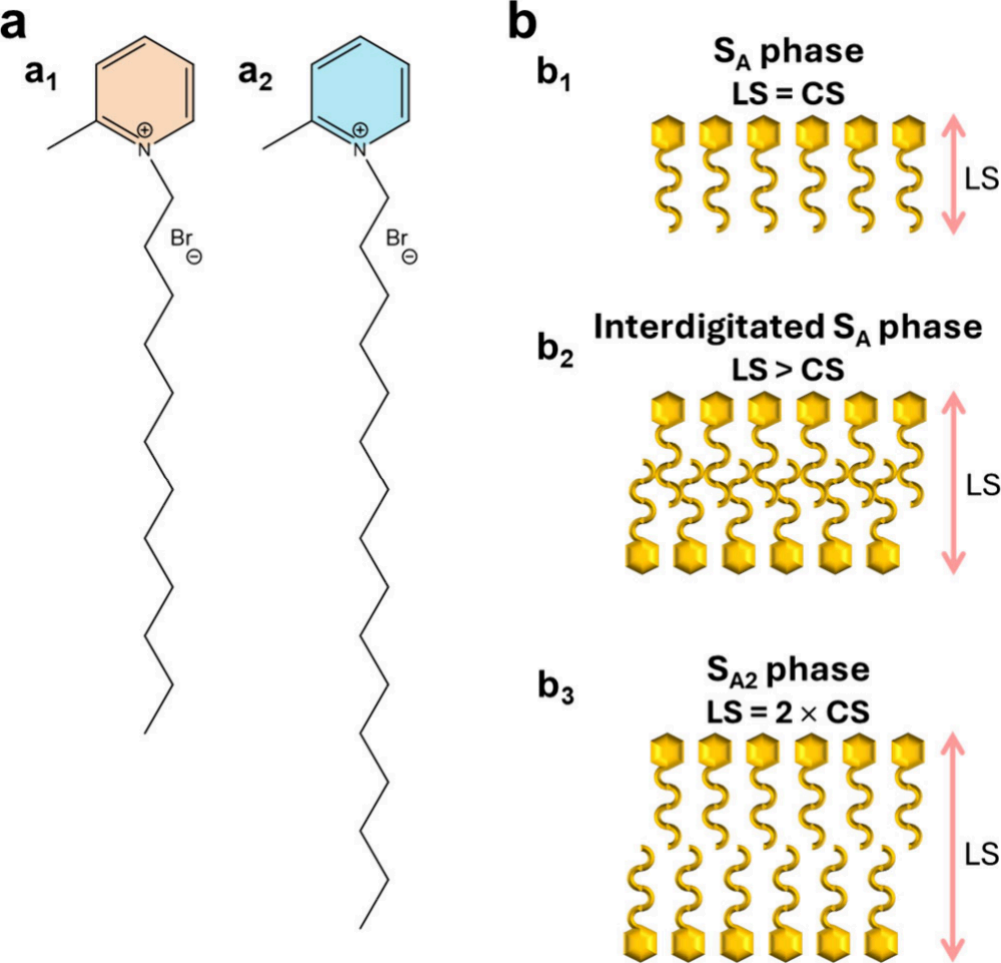
(a) Chemical structures: (a_1_) [C_12_-2-Pic][Br] and (a_2_) [C_16_-2-Pic][Br]. (b) Scheme of the liquid crystalline smectic phases: (b_1_) smectic A (S_A_), (b_2_) interdigitated S_A_ bilayer, and (b_3_) totally separated S_A_ bilayer (S_A2_). LS means layer spacing, whereas CS refers to the cation size.

Aiming to understand the self-assembly and the phase structure of the developed lyotropic systems, all prepared solutions were characterized by polarized optical microscopy (POM) and X-ray powder diffraction (XRD). Figure 1 comprises the microphotographs and the diffractograms collected for (a) [C_12_-2-Pic][Br]_aq_ and (b) [C_16_-2-Pic][Br]_aq_. Furthermore, the chemical structures of the studied compounds and the scheme illustrating the liquid crystalline smectic phases addressed in this work are listed in Figure 2.

For a 60% mixture of [C_12_-2-Pic][Br]_aq_ (Figure 1a, top), the observation of birefringence and fluidity at room temperature indicates the presence of a lyotropic mesophase. Moreover, the texture detected by POM, focal conics, suggests a lamellar structural organization, later confirmed by XRD. Regarding [C_16_-2-Pic][Br]_aq_ 40% (Figure 1b, top), after shearing, birefringent regions also emerged, although no specific texture was identified.

The accurate determination of the critical concentration, as well as the structural arrangement addressed to each lyotropic system, was performed by XRD. For [C_12_-2-Pic][Br]_aq_ (Figure 1a, bottom), it was found one peak at small angles arising between 20 and 30 wt %, while, for [C_16_-2-Pic][Br]_aq_ (Figure 1b, bottom), this scattering signature appears around 20 wt %. In both systems, the coexistence of an intense peak at small angles with no evidence of other peaks at large angles points to the existence of a disordered lamellar phase. Nonetheless, the broadness of the diffractogram’s peak is also compatible with an isotropic phase comprising organized domains. In fact, thermotropic mesophases were previously found for both ILCs at neat conditions, being defined as smectic phases.^26,27^ Curiously, while bulk [C_16_-2-Pic][Br] is monotropic on heating, displaying a S_A_ phase, the shorter salt, [C_12_-2-Pic][Br], exhibits two different liquid crystalline phases: S_1_, i.e., undetermined ordered lamellar phase, on heating, and S_A_, upon cooling.^27^ However, the layer spacing estimated for the lyotropic [C_12_-2-Pic][Br]_aq_ (Figure S1) indicates that the water promotes an increase of the layer spacing, translating into a higher separation of the bilayer, although still being interdigitated (Figure 2). In order to evaluate the thermal stability of this phase, further diffractograms of the highest concentration (60 wt %) were collected upon temperature (Figure S2). The diffractograms denote a well-developed smectic A phase, revealing the same structure from 23 to 50 °C. For [C_16_-2-Pic][Br]_aq_, the water has no impact on the self-assembly, since the lyotropic liquid crystalline phase matches the thermotropic one at all concentrations, as summarized in Figure S1 and Table 1.

**Table 1:** Cation Size and Layer Spacing Values Obtained for the Thermotropic and Lyotropic Aqueous Phases of [C_12_-2-Pic][Br] and [C_16_-2-Pic][Br]

		layer spacing (Å)	
	cation size^a^ (Å)	thermotropic phase^b^	lyotropic phase^c^
[C_12_-2-Pic][Br]	20.0	23.8 (S_1_); 24.1 (S_A_)	34.1 (S_A_)
[C_16_-2-Pic][Br]	25.0	33.1 (S_A_)	34.8 (S_A_)

a Cation size estimated by Avogadro molecular modeling software (version: 1.2.0).

b Values of the neat thermotropic phases taken from refs (26) and (27).

c Values correspond to the layer spacing obtained for [C_12_-2-Pic][Br]_aq_ 60% and for the average of [C_16_-2-Pic][Br]_aq_ 20% and 30%. Figure S1 comprises the real values of each concentration above the lamellar critical point with the corresponding errors.

This section allowed us to conclude that both aqueous systems exhibit liquid crystalline properties at room temperature, for which S_A_ phases were found for [C_12_-2-Pic][Br]_aq_ and [C_16_-2-Pic][Br]_aq_. Therefore, [C_12_-2-Pic][Br] and [C_16_-2-Pic][Br] can be designated as amphotropic ionic liquid crystals, possessing thermotropic and lyotropic mesophases.

### Molecular Dynamics of Lyotropic Systems

**Figure 3:**
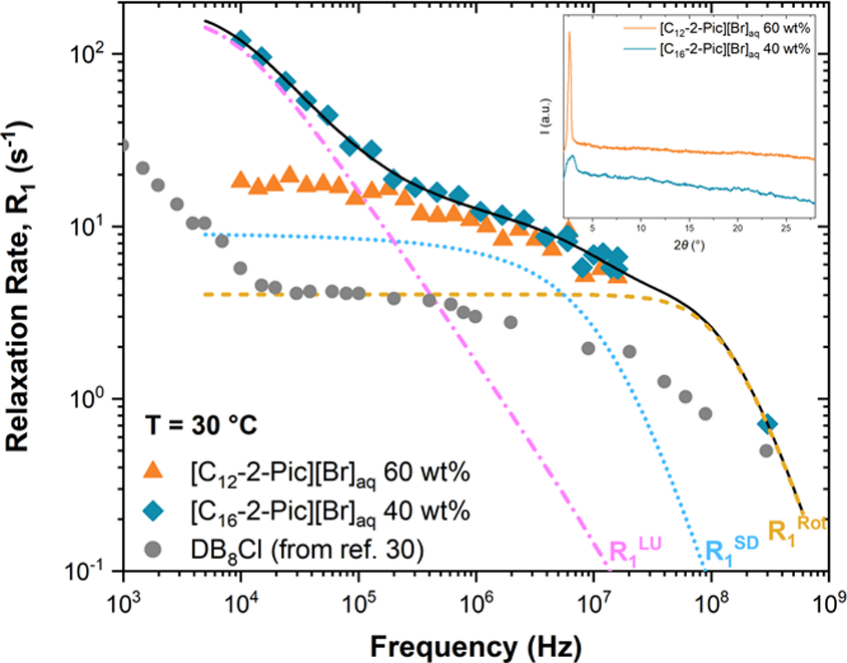
Frequency dispersion of the relaxation rate in the S_A_ phase of [C_12_-2-Pic][Br]_aq_ 60% (triangles) and [C_16_-2-Pic][Br]_aq_ 40% (diamonds) compared with the DB_8_Cl (circles) data^30^ for the same phase. Solid line represents the best model fitting curve, *R*_1_, to the experimental data obtained for [C_16_-2-Pic][Br]_aq_ with eq 1, while dotted and dashed lines correspond to the relaxation contributions *R*_1_^LU^, *R*_1_^SD^, and *R*_1_^Rot^. The inset shows the XRD collected at the same temperature of the NMR assays (30 °C).

In the previous section, XRD suggested a lamellar structure for the aqueous system formed by [C_16_-2-Pic][Br]_aq_. Nonetheless, the peak collected is also coherent with an isotropic phase containing some local organized domains. Additionally, for [C_12_-2-Pic][Br]_aq_, a smectic A phase was found for concentrations above 20 wt %. Therefore, to confirm and clarify the phase structure of both lyotropic systems, molecular dynamics were assessed by nuclear magnetic resonance, in particular by fast field cycling (FFC-NMR), through the determined NMR relaxation rates. Figure 3 presents the spin–lattice relaxation rate, as a function of frequency, obtained for [C_12_-2-Pic][Br]_aq_ and [C_16_-2-Pic][Br]_aq_ lyotropic systems at the highest concentrations prepared: 60 and 40 wt %, respectively. For comparison purposes, Figure 3 also includes the relaxation dispersion found for the bilayer smectic A_2_ phase of the liquid crystal 4-octylphenyl 2-chloro-4-(4-cyanobenzoyloxy)benzoate (DB_8_Cl).^30^

The *R*_1_ obtained for [C_16_-2-Pic][Br]_aq_ 40% (Figure 3, diamonds) represents the sum of the mechanisms mentioned in the Experimental Part: local molecular rotations (Rot), self-diffusion (SD), and layer undulations (LU). It is known that their presence explains the relaxation rate in lamellar structures. Moreover, the fact that a very clear component of layer undulations is detected at frequencies below 10^5^ Hz points to a S_A_ phase. This is also in agreement with the similarity between the *R*_1_ dispersions of [C_16_-2-Pic][Br]_aq_ 40% and the S_A_ phase of the liquid crystal DB_8_Cl.^30^ Hence, these preliminary studies endorse the formation of a lyotropic system for [C_16_-2-Pic][Br]_aq_.

Contrarily, an almost invariant dispersion was registered for [C_12_-2-Pic][Br]_aq_ 60% (Figure 3, triangles) in the low frequency range. Taking into account that POM and XRD studies showed evidence of a lamellar structure, it should be expected to observe an increase in *R*_1_ associated with the layer undulations contribution of this compound. Since our measurements were limited to frequencies above 10^4^ Hz due to experimental limitations of the currently available FFC-NMR equipment, it is possible that such relaxation enhancement occurs below 10^4^ Hz. In fact, the DB_8_Cl R_1_ dispersion reveals a strong increase in this particular frequency range. In this context, it is possible to infer that the lyotropic [C_12_-2-Pic][Br]_aq_ might present a lamellar structure with a coherence length higher than the one of [C_16_-2-Pic][Br]_aq_, which is in line with the sharped and narrow XRD peak observed at low angles for the shorter salt (see the inset of Figure 3).

It is worthwhile mentioning that the spin–lattice relaxation is quite sensitive to different levels of local molecular organization, orientational and/or lamellar, even in the case of nonliquid crystal materials.^32^ Nevertheless, NMR measurements support the hypothesis raised by POM and XRD of a smectic phase present in both lyotropic systems, emphasizing the induction of mesomorphic behavior at operating temperatures.

### Critical Micelle Concentration and Foam Formation

**Figure 4:**
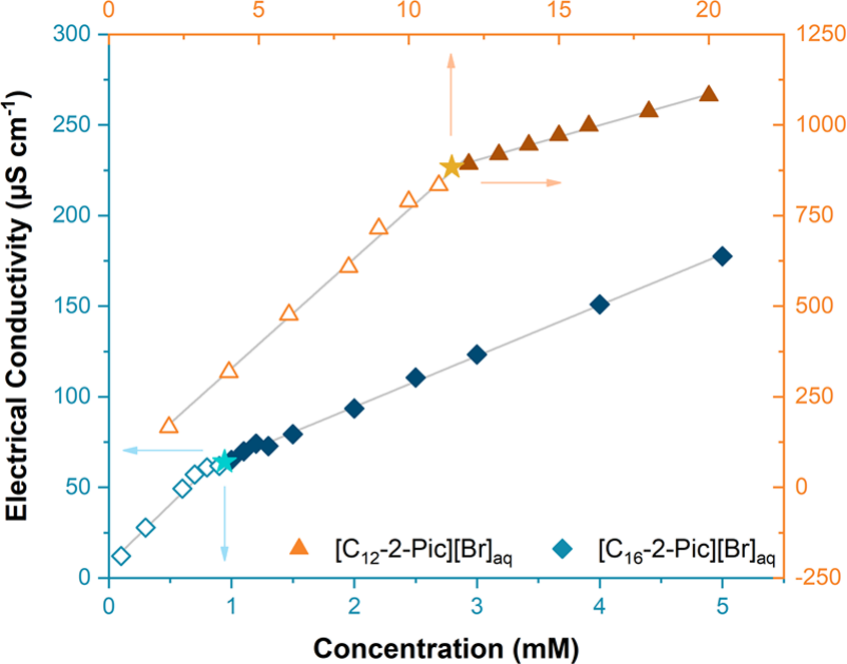
Micellization behavior obtained for [C_12_-2-Pic][Br]_aq_ (triangles) and [C_16_-2-Pic][Br]_aq_ (diamonds). Open and full symbols correspond to the points collected below and above the critical micelle concentration, respectively, whereas the star-shaped symbols represent the exact values of the CMCs: 11.4 and 0.9 mM.

In order to study the micellization behavior of [C_12_-2-Pic][Br]_aq_ and [C_16_-2-Pic][Br]_aq_, the critical micelle concentration (CMC) was determined by electrical conductivity for both materials. This technique can be applied exclusively on surfactants whose nature is ionic,^33^ as the studied ionic liquid crystals, since the nonionic molecules are not capable of changing the conductivity in solution.^34^ Therefore, for each compound, several aqueous mixtures were prepared with different concentrations and then measured. Figure 4 illustrates the concentration profile against the electrical conductivities for [C_12_-2-Pic][Br]_aq_ and [C_16_-2-Pic][Br]_aq_. The respective values used to obtain the curve and extrapolate the minimal concentration above which occurs the formation of micelles are listed in Table S1.

Regarding [C_12_-2-Pic][Br]_aq_ (Figure 4, triangles), two different linear dependences between the concentration and conductivity are observed. Below the CMC, the progressive addition of [C_12_-2-Pic][Br] translates into an abrupt increase of conductivity due to the higher amount of dissolved cations ([C_12_-2-Pic]^+^) and anions ([Br]^−^) in solution. After reaching the minimal concentration to form micelles spontaneously (see the star-shaped symbol in Figure 4), [C_12_-2-Pic][Br] added to the system acts as a surfactant, originating foams, which leads to a less pronounced rise of conductivity as well as a smaller linear slope.

Likewise, a similar behavior is detected for [C_16_-2-Pic][Br]_aq_ (Figure 4, diamonds). However, the CMC extrapolated (0.9 mM) is 10-fold lower than the one obtained for [C_12_-2-Pic][Br]_aq_ (11.4 mM). This can be explained with the length of alkyl chain. Since the hydrophobic character is directly related to the size of the aliphatic group, molecules with longer chains exhibit lower water solubility and, thus, a lower CMC.

The dispersion profile obtained for [C_12_-2-Pic][Br]_aq_ and [C_16_-2-Pic][Br]_aq_ shows that both materials have surfactant properties, allowing them to be classified as cationic surfactants and foam formers. Moreover, the two CMC determined in this work are in accordance with the values reported for the pyridinium bromide derivatives [C_12_Pyr][Br] (12 mM^35^) and [C_16_Pyr][X] (X = Br^–^ or Cl^–^; 0.6–1.1 mM^36,37^). This suggests that the incorporation of the methyl group at *ortho-* position does not impact significantly the micellization behavior of the studied compounds compared to the unsubstituted materials.

### Foam Studies and Kinetics

**Figure 5:**
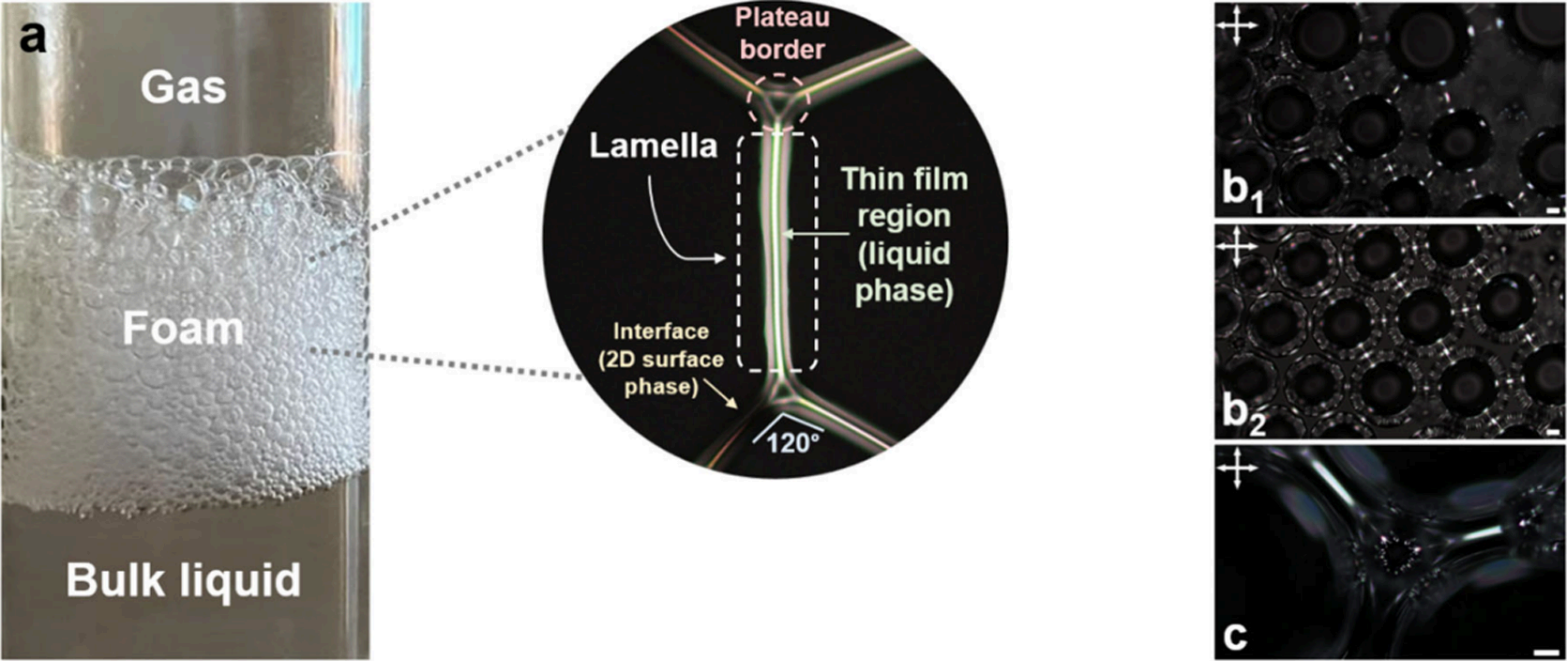
General foam scheme of the diluted [C_16_-2-Pic][Br]_aq_ 1.3 mM in water: (a) illustration of the three regions existing in a liquid foam with the corresponding nomenclature assigned for the magnified two-dimensional slice, (b) sequence of microphotographs, showing the effect of water drainage, and (c) detail at the boundary of the bubbles. Parts b and c were taken in transmission mode, under crossed polarizers, and without coverslip. The scale bars indicate 50 μm.

**Figure 6:**
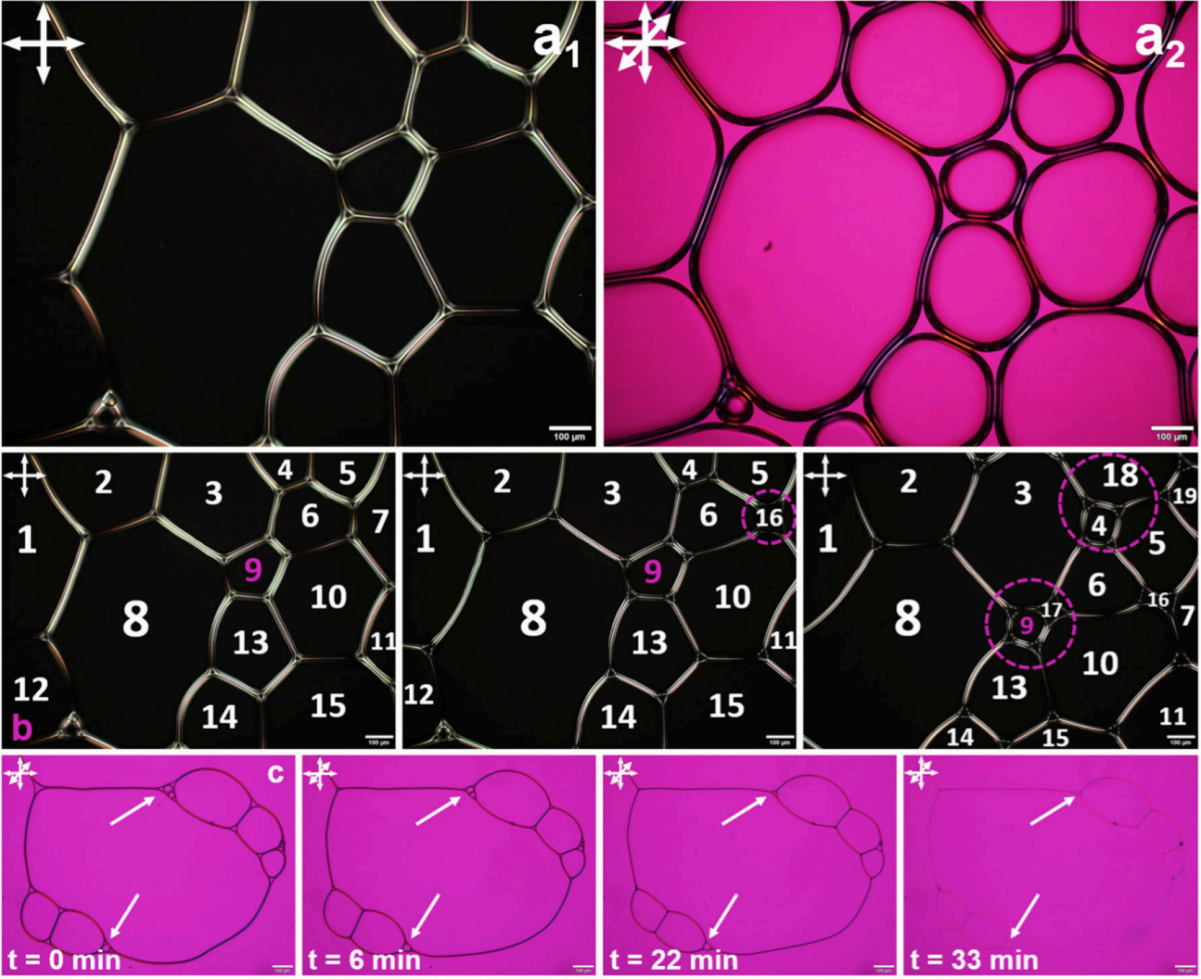
Optical microphotographs of wet 2D foams of (a) diluted [C_16_-2-Pic][Br]_aq_ 0.9 mM in water. (b) Sequence of pictures for the same system, evidencing two different transformations (T_r_): T_r1_, cells 6 and 7 cease to be neighbors via the appearance of the 4-fold vertex instable cell 16, and T_r2_, the shrinking of a bubble and its disappearance (e.g., cell 9). (c) Sequential photos of the diluted [C_12_-2-Pic][Br]_aq_ 4 mM in water, emphasizing the presence of a wet foam and the disappearance of the bubbles (arrows). Parts a_1_ and b were collected between cross-polarizers, while parts a_2_ and c were acquired between cross-polarizers and a lambda plate (λ = 530 nm). The scale bars indicate 100 μm.

Foam analysis relied on the films created after shearing the diluted aqueous mixtures, i.e., isotropic solutions, of both ILCs above the respective CMC. Figure 5 displays the general foam system observed for the lengthiest salt ([C_16_-2-Pic][Br]_aq_). Afterward, the two preparations were observed under POM between crossed polarizers and between crossed polarizers with a retardation waveplate (λ = 530 nm) introduced at 45° to enhance the contrast of the anisotropic boundaries of the foams. Moreover, preliminary studies regarding the kinematics of the systems were also performed, as illustrated in Figure 6.

The general foam structure, which appears between the liquid at the bottom and the gas phase at the top, is depicted in Figure 5a. In the magnified image, the different parts of the foam structure are emphasized. The lamella is defined as the region that comprises the birefringent thin film and its two interfaces on either side as well as part of the junction to other lamellae. Moreover, the connection of three lamellae is designated as a plateau border, possessing an angle of 120°. Figures 5b_1_ and 5b_2_ were taken at two different sequential times, showing the reorganization and the increased contact area between the bubbles. The magnification of Figure 5a represents a two-dimensional slice due to a coverslip, different from that in Figure 5c. In this figure, where no coverslip was used, the 3D out-of-plane extension of the plateau borders led to the difficulty of focusing only one plane of the microscopic preparation.

For the diluted [C_16_-2-Pic][Br]_aq_ 0.9 mM system, Figures 6a_1_ and 6a_2_ denote the 2D foam structure with isotropic air regions, i.e., large black and pink domains on the left and right images, respectively, surrounded by birefringent walls, all with the same average thickness of about 27 μm. The structure of the plateau borders can also be detected, having triangular isotropic shapes contoured by birefringent films. Once again, the internal angles are around 120°, as expected for an equilibrium liquid foam with uniform film tension.^18^ Furthermore, in Figure 6a_2_, the blue and pink colors, which appear at different angles, indicate that the anisotropic fluid orientates along the walls of the lamellas. On the other hand, bubbles with more than six edges, such as numbers 8 and 10 in Figure 6b, increase in area, whereas cells with fewer edges, as 6 and 7, cease to be neighbors via the appearance of a 4-fold vertex instable (cell 16).

Moreover, Figure 6c reveals that birefringent foams are also registered for the [C_12_-2-Pic][Br]_aq_ 4 mM diluted system, even below the CMC value. In a time frame of 30 min, it was possible to see that cells with less than six sides shrink and disappear, as pointed by the arrows and also observed for [C_12_MiM][Br].^24,25^

Kinetic-wise, foams made from [C_16_-2-Pic][Br]_aq_ solutions are more stable, i.e., last longer in time, than those of [C_12_-2-Pic][Br]_aq_, which can be explained by the longer alkyl chain that promotes higher foam stability. In general, the viscosity of ionic liquids depends on the size of both moieties, being lower for larger species and strongly correlated to the anion. However, for cations, it is known that the lengthening of the alkyl chain implies an increase in viscosity due to stronger van der Waals interactions.^38^ Another parameter that varies with the size of the aliphatic group is the surface tension. In fact, Coutinho et al.^39^ reported that increasing the cation size up to hexyl of two imidazolium families led to a decrease in the surface tension. At the same time, it remained almost invariant for the dodecyl and hexadecyl chains. Therefore, the observed behavior could indicate that elasticity forces are also playing a role in the foam stability^40^ of [C_12_-2-Pic][Br]_aq_ and [C_16_-2-Pic][Br]_aq_.

## Conclusions

In this work, a simple strategy was explored to bring the mesomorphic behavior of two thermotropic ionic liquid crystals to room temperature: [C_12_-2-Pic][Br] and [C_16_-2-Pic][Br]. In this context, lyotropic aqueous systems were prepared, and the phases above and below the critical concentration were investigated. For [C_12_-2-Pic][Br]_aq_, POM microphotographs evidence the presence of a birefringent texture and more specific focal conics, characteristic of smectic phases. The scattering profile obtained by X-ray powder diffraction corroborates the lamellar structure, emerging between 20 and 30 wt %. Moreover, the water promoted an increase of the layer spacing, translating into a higher separation of the bilayer, although still being interdigitated, even for the highest concentration (60 wt %) where the diffractogram points to a well-developed smectic A phase. The thermal stability of the developed lyotropic system was evaluated, revealing the same structure up to 50 °C. On the other side, the amphotropic character of [C_16_-2-Pic][Br] was more difficult to investigate, as the diffractograms recorded are suggestive of either an isotropic phase bearing some local organized domains or a lamellar structure with a short correlation length. Nonetheless, the critical concentration of this liquid crystalline phase is around 20 wt %. Regarding the phase structure, the scattering signature, along with the determined layer spacing, suggests an interdigitated smectic A phase, which matches the one of neat [C_16_-2-Pic][Br]. In order to confirm the existence of a lamellar structure, the molecular dynamics of [C_16_-2-Pic][Br]_aq_ 40% was assessed by fast field cycling nuclear magnetic resonance. The relaxation rate obtained for this mixture exhibited a strong component of layer undulations, which are characteristic of smectic phases, corroborating the XRD results.

Furthermore, several isotropic diluted solutions were prepared for [C_12_-2-Pic][Br]_aq_ and [C_16_-2-Pic][Br]_aq_, allowing the determination of the critical micelle concentration: 11.4 mM for the former and 0.9 mM for the latter. The surfactant nature of both salts resulted in shear-induced foams, which were studied in terms of morphology and kinetics. It was found that both systems present birefringent liquid foams below and above the CMC value. Moreover, the disappearance of the cells follows the laws for wet foams. These initial results also indicate that foams derived from [C_16_-2-Pic][Br]_aq_ are more stable than those of [C_12_-2-Pic][Br]_aq_, which can be explained by the longer alkyl chain that imprints higher viscosity and, thus, increased foam stability.

The insights presented on aqueous lyotropic liquid phases and birefringent foams, both from the same ionic liquid crystals, could be relevant for future applied studies, opening new horizons regarding the shift to room temperature of mesomorphic behavior that appears at high temperatures and the tuning of foam stability by varying the alkyl chain length.

## Supporting Information Available

Diffractograms collected upon temperature for the lyotropic [C_12_-2-Pic][Br]_aq_, as well as the layer spacing values of both systems and the replicates used to determine the critical micelle concentration (PDF)
