# Supplementary material for: Lyotropic Aqueous 2-Picolinium Ionic Liquid Crystals and Their Shear-Induced Foams
Source: Langmuir. 2024 Aug 17;40(38):19964–71. doi: 10.1021/acs.langmuir.4c02059 (PMC11448070; doi:10.1021/acs.langmuir.4c02059)
Supplement: Supplementary file 1 — la4c02059_si_001.pdf [file la4c02059_si_001.pdf]

Supporting Information

Lyotropic Aqueous 2-Picolinium Ionic Liquid Crystals and Their Shear-Induced Foams

Andreia F. M. Santos [a], Anton Gradišek [b], Tomaž Apih [b], Pedro J. Sebastião [c], Madalena Dionísio [a], Luis C. Branco [a], João L. Figueirinhas [c]* and Maria H. Godinho [d]*

[a] *LAQV-REQUIMTE, Department of Chemistry, NOVA School of Science and Technology, NOVA University of Lisbon, Campus de Caparica, 2829-516 Caparica, Portugal;*
[b] *Jožef Stefan Institute, Jamova Cesta 39, 1000, Ljubljana, Slovenia;*
[c] *CeFEMA and Department of Physics, Instituto Superior Técnico, University of Lisbon, Av. Rovisco Pais, 1, 1049-001 Lisbon, Portugal;*
[d] *i3N/CENIMAT, Department of Materials Science, NOVA School of Science and Technology, NOVA University of Lisbon, Campus de Caparica, 2829-516 Caparica, Portugal.*

*Corresponding authors: joao.figueirinhas@tecnico.ulisboa.pt and mhg@fct.unl.pt

Mesomorphic behaviour of lyotropic systems:

Aiming to understand the liquid crystal properties at room temperature, both lyotropic systems were characterised by Polarised Optical Microscopy (POM) and X-Ray Powder Diffraction (XRD). Figure S1 displays the obtained layer spacings in function of concentration, whereas Figure S2 comprises the diffractograms collected upon heating for the lyotropic [C_{12}-2-Pic][Br]$_{aq}$ 60 wt%, allowing to evaluate its thermal stability.

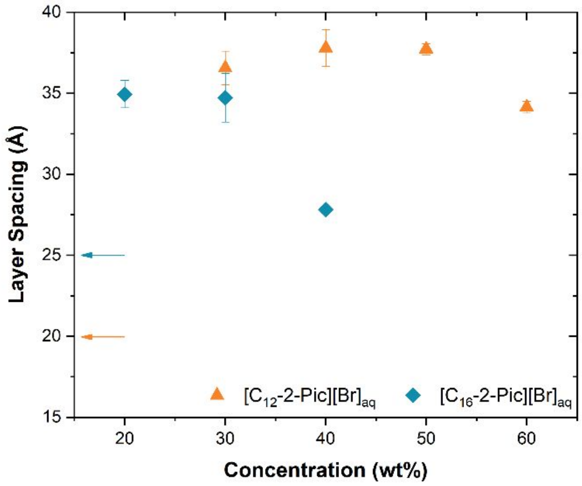

Figure S1 – Layer spacing values obtained for [C_{12}-2-Pic][Br]$_{aq}$ (triangles) and [C_{16}-2-Pic][Br]$_{aq}$ (diamonds). The arrows indicate the corresponding cation size, being 20 Å for the former and 25 Å for the latter.

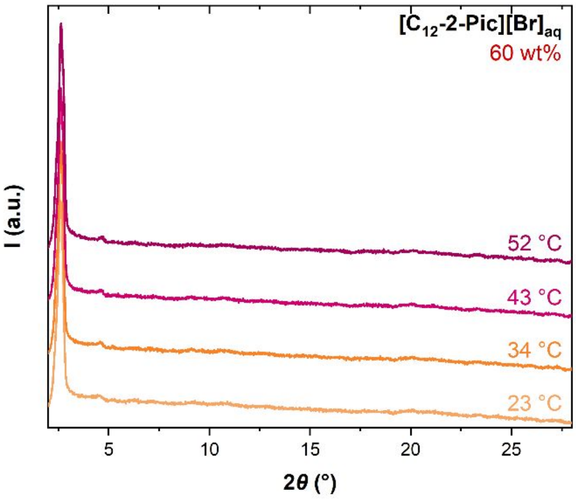

Figure S2 – Diffractograms collected for [C_{12}-2-Pic][Br]$_{aq}$ 60 wt% upon heating, from 23 to 52 °C, which confirms the high thermal stability of the lyotropic phase structure.

Critical micelle concentration and foam formation:

CMC values of [C_{12}-2-Pic][Br]$_{aq}$ and [C_{16}-2-Pic][Br]$_{aq}$ were extrapolated by the electrical conductivity assays. In this context, several solutions with different concentrations were prepared and Table S1 comprises the data collected, as well as the water conductivity, which was used as reference. Both micellization profiles were built using the average of the four independent electrical conductivities acquired for each solution.

Table S1 – Data collected to determine the CMC values of [C_{12}-2-Pic][Br]$_{aq}$ and [C_{16}-2-Pic][Br]$_{aq}$.

	Concentration	Electrical conductivity (µS cm^{-1})				
	(mM)	**Exp. 1**	**Exp. 2**	**Exp. 3**	**Exp. 4**	**AVERAGE**
**H_2O**	-	1.27				
**[C_{12}-2-Pic][Br]$_{aq}$**	**2**	166.0	166.1	166.1	166.2	**166.1**
	**4**	317	318	318	319	**318**
	**6**	472	476	478	480	**476.5**
	**8**	603	607	610	612	**608**
	**9**	712	714	715	716	**714.25**
	**10**	788	789	790	790	**789.25**
	**11**	834	833	833	834	**833.5**
	**12**	890	893	893	893	**892.25**
	**13**	918	918	919	919	**918.5**
	**14**	944	945	945	945	**944.75**
	**15**	969	972	972	971	**971**
	**16**	997	998	998	998	**997.75**
	**18**	1035	1037	1038	1038	**1037**
	**20**	1077	1081	1082	1083	**1080.75**
**H_2O**	-	2.26				
**[C_{16}-2-Pic][Br]$_{aq}$**	**0.1**	12.08	12.07	12.04	12.01	**12.05**
	**0.3**	27.8	27.8	27.8	27.8	**27.8**
	**0.6**	49.2	49.4	49.4	49.4	**49.35**
	**0.7**	57.0	57.1	57.2	57.2	**57.125**
	**0.8**	60.8	61.0	61.1	61.2	**61.025**
	**0.9**	61.8	61.9	62.0	62.0	**61.925**
	**1**	64.8	65.0	65.2	65.3	**65.075**
	**1.1**	69.8	69.8	69.9	70.0	**69.875**
	**1.2**	73.8	74.2	74.2	74.2	**74.1**
	**1.3**	72.4	72.8	72.9	73.0	**72.775**
	**1.5**	79.1	79.4	79.5	79.5	**79.375**
	**2**	93.3	93.5	93.6	93.7	**93.525**
	**2.5**	110.3	110.5	110.6	110.6	**110.5**
	**3**	123.1	123.3	123.4	123.4	**123.3**
	**4**	150.8	151.0	151.0	151.1	**150.975**
	**5**	177.3	177.5	177.6	177.7	**177.525**
